# TNF-Alpha as an Initiator of Allodynia and Anxiety-Like Behaviors in a Preclinical Model of PTSD and Comorbid Pain

**DOI:** 10.3389/fpsyt.2021.721999

**Published:** 2021-08-25

**Authors:** Patrick Dib, Yong Zhang, Michael A. Ihnat, Randle M. Gallucci, Kelly M. Standifer

**Affiliations:** ^1^Department of Pharmaceutical Sciences, University of Oklahoma College of Pharmacy, University of Oklahoma Health Sciences Center, Oklahoma City, OK, United States; ^2^Harold Hamm Diabetes Center, College of Medicine, University of Oklahoma Health Sciences Center, Oklahoma City, OK, United States; ^3^Department of Physiology, College of Medicine, University of Oklahoma Health Sciences Center, Oklahoma City, OK, United States; ^4^Department of Cell Biology, College of Medicine, University of Oklahoma Health Sciences Center, Oklahoma City, OK, United States

**Keywords:** hyperalgesia, allodynia, nociceptin/orphanin FQ (N/OFQ), traumatic stress, thalidomide, NOP receptor, anti-TNF alpha antibody

## Abstract

Post-Traumatic Stress Disorder (PTSD) is a debilitating mental health disorder that occurs after exposure to a traumatic event. Patients with comorbid chronic pain experience affective distress, worse quality of life, and poorer responses to treatments for pain or PTSD than those with either condition alone. FDA-approved PTSD treatments are often ineffective analgesics, requiring additional drugs to treat co-morbid symptoms. Therefore, development of new treatment strategies necessitate a better understanding of the pathophysiology of PTSD and comorbid pain. The single prolonged stress (SPS) model of PTSD induces the development of persistent mechanical allodynia and thermal hyperalgesia. Increased Nociceptin/Orphanin FQ (N/OFQ) levels in serum and CSF accompany these exaggerated nociceptive responses, as well as increased serum levels of the pro-inflammatory cytokine tumor necrosis factor (TNF-α). Therefore, the primary goal was to determine the role of TNF-α in the development of SPS-induced allodynia/hyperalgesia and elevated serum and CNS N/OFQ using two approaches: TNF-α synthesis inhibition, and blockade with anti-TNF-α antibody that acts primarily in the periphery. Administration of TNF-α synthesis blocker, thalidomide (THL), immediately after SPS prevented increased TNF-α and development of allodynia and hyperalgesia. The THL effect lasted at least 21 days, well after thalidomide treatment ended (day 5). THL also prevented SPS-induced increases in serum N/OFQ and reversed regional N/OFQ mRNA expression changes in the CNS. Serum TNF-α increases detected at 4 and 24 h post SPS were not accompanied by blood brain barrier disruption. A single injection of anti-TNF-α antibody to male and female rats during the SPS procedure prevented the development of allodynia, hyperalgesia, and elevated serum N/OFQ, and reduced SPS-induced anxiety-like behaviors in males. Anti-TNFα treatment also blocked development of SPS-induced allodynia in females, and blocked increased hypothalamic N/OFQ in males and females. This suggests that a peripheral TNF-α surge is necessary for the initiation of allodynia associated with SPS, as well as the altered central and peripheral N/OFQ that maintains nociceptive sensitivity. Therefore, early alleviation of TNF-α provides new therapeutic options for investigation as future PTSD and co-morbid pain treatments.

## Introduction

Chronic or persistent pain is one of the most commonly co-occurring physical problems for patients with PTSD, and an even higher incidence of this comorbidity was observed in the veteran population (1). Further complicating this co-morbidity are findings that patients with chronic pain and PTSD experience more intense pain and affective distress, higher levels of life interference, and greater disability than patients with either condition alone (2–4). Unfortunately, the trigger for development of PTSD and co-morbid pain symptoms is unknown, but findings by us and others using the single prolonged stress (SPS) model of PTSD suggest that tumor necrosis factor-α (TNF-α) initiates the development of pain and anxiety-like behaviors. Critically, this has not been tested directly. Nociceptin/Orphanin FQ (N/OFQ) is an opioid peptide that binds to fourth member of the opioid receptor superfamily, the N/OFQ peptide (NOP) receptor (5). N/OFQ and the NOP receptor are located in brain regions and nerve endings mediating pain sensitivity (6). Peripheral release of N/OFQ increases macrophage and monocyte infiltration, contributing to inflammation and nociceptive hypersensitivity (7). Therefore, because N/OFQ can bi-functionally modulate pain sensitivity, it is important to understand how N/OFQ is modulated under conditions that increase pain sensitivity and allodynia.

The single prolonged stress (SPS) model of PTSD has been employed as a preclinical model of PTSD for over 20 years (8, 9). Previous work in our lab and that of others has shown that SPS induces the development of persistent mechanical allodynia and thermal hyperalgesia (10), visceral hypersensitivity (11) and stress- (12) and surgically-induced hypersensitivity (13, 14). Appearance of increased nociceptive sensitivity in this model paralleled increased TNF-α acutely in serum (14–16). Subsequent to development of allodynia and hyperalgesia, N/OFQ levels increased in serum, CSF, PAG, hippocampus, and hypothalamus (10, 17, 18). Our lab demonstrated that mechanical allodynia and thermal hyperalgesia induced by SPS lasts at least 30 days in male and female rats, and is blocked by NOP receptor antagonists or absence of the NOP receptor (10, 17, 18). Interestingly, TNF-α increases N/OFQ expression (19, 20). Elevated circulating TNF-α has been noted in PTSD patients (including non-combat related trauma patients) (21, 22). TNF-α production in peripheral blood mononuclear cells (PBMCs) from PTSD patients was increased in response to LPS (23), compared to cells from non-PTSD control subjects, suggesting sensitization of the immune response with PTSD. The caveat of this comparison is that clinical data may be collected months-years after the initial traumatic event, so the timing of the increase in TNF-α differs from experimentally derived data. Further, the frequency and severity of PTSD symptoms correlates with circulating levels of several different cytokines besides TNF-α, including IL-1β (21, 22).

Anti-TNF-α therapy in rheumatoid arthritis patients decreases brain-derived TNF-α and alleviates pain (24). Consequently, drugs that reduce TNF-α synthesis may reduce pain, anxiety and depressive symptoms by moderating TNF-α-induced changes in neurotransmission (25). Reduced hyperalgesia and/or anxiety behaviors following SPS correlated with reduced circulating and hippocampal TNF-α and other inflammatory cytokines 1–2 weeks post-SPS (13, 26). However, those studies did not directly target TNF-α synthesis or activity. The primary goal of this study was to test the hypothesis that blockade of TNF-α synthesis or action shortly after initiation of a traumatic stressor (SPS) would prevent development of mechanical (tactile) allodynia, thermal hyperalgesia and subsequent elevation of N/OFQ. A secondary goal of the study was to evaluate anxiety-like behaviors.

## Materials and Methods

### Animals

Adult Sprague–Dawley rats weighing 220–250 g at the initiation of SPS were obtained from Charles River Labs (Wilmington, MA). Animals were housed in the animal facility under a 12-h light: 12-h dark cycle (lights on at 06:00 h) with free access to food and water. After arrival, rats were acclimated to the animal facility for 7–10 days prior to initiation of experiments. Experimental protocols were approved by the Institutional Animal Care and Use Committee of the University of Oklahoma Health Sciences Center and the US Army Medical Research and Materiel Command Animal Care and Use Review Office. Research was compliant with the Animal Welfare Act Regulations and other Federal Statutes relating to animals and experiments involving animals, and adhered to the principles set forth in the Guide for Care and Use of Laboratory Animals, National Research Council, 1996. All experiments conformed to the guidelines of the International Association for the Study of Pain. Every effort was made to minimize animal discomfort and reduce the number of animals used.

### SPS

Animals were randomized into groups. The SPS procedure was followed as described (8, 27) with modification (10). After acclimatization, rats were exposed to complete restraint in disposable plastic holders for 2 h, followed by grouped (3–4 rats) forced swimming for 20 min in a cylindrical plexiglass tank (46 cm tall × 20 cm in diameter) filled with 22°C water to a depth of 30 cm. After a 15 min recovery and drying period, animals were exposed to diethyl ether in a fume hood until consciousness was lost. Upon awakening, rats were returned to their cages for the rest of the study.

### Thalidomide (THL) Treatment

The THL dose effectively reduced TNF-α synthesis (28). THL (50 mg/kg dissolved in 2% DMSO in saline) or vehicle alone was injected intraperitoneally (0.3 cc vol) into male rats approximately 1–2 h following recovery from ether anesthesia, and daily thereafter at the same time of day, for a total of 5 injections. Rats were euthanized at day 9 (*n* = 6/group) or 21 (*n* = 3/group), 9 total rats per group were assessed for nociceptive sensitivity on days 0, 3, 7 and 9 (for all 9 rats/group). Four groups of three rats/group also were tested on days 13 and 21 as a pilot experiment to determine if latent allodynia or hyperalgesia appeared over the subsequent 12 days.

### Anti-TNF-α Antibody Treatment

Male and female Sprague-Dawley rats (200~225 g) were randomly divided into 4 groups: control + IgG, control + anti-TNF-α, SPS + IgG and SPS + anti-TNF-α (N = 5~6/group/sex). Anti-TNF-α antibody (30 μg) (29) or the same amount of normal goat IgG were injected during the second hour of restraint in SPS or control rats.

### Nociceptive Sensitivity Tests

Rats were assessed changes in nociceptive responses to tactile and thermal stimuli in all groups after placement in clear plastic boxes with a glass floor for thermal tests and a wire mesh floor for tactile assessments. Animals acclimated to the boxes for 15–30 min prior to assessment. A plantar analgesia meter (IITC Life Science Inc., Woodland Hills, CA) was utilized to measure paw withdrawal latency (PWL) to an infrared light beam (thermal sensitivity) directed toward the right hind paw with the lamp set at 25% active intensity. Cut-off time was set at 30 s to prevent tissue damage (30). An Electronic von Frey anesthesiometer (IITC Life Science, Inc., Woodland Hills, CA) was utilized for tactile (mechanical) sensitivity assessment. Paw withdrawal thresholds (PWT) from the von Frey-like stimuli were obtained from the mid-plantar aspect of the right hind paw. The responses to thermal and tactile stimuli were tested 2 h apart. The average of 3 assessments spaced 5 min apart were compared between groups for each test. SPS began at least 1 h after baseline pain thresholds were assessed.

### Elevated Plus Maze (EPM) Test

Rats were tested on the EPM on day 9 after SPS for the appearance of anxiety-like behaviors (31). EPM tests occurred between 09:00 and 10:30 h, before nociceptive sensitivity assessment. The plus maze consisted of two open (50 × 10 cm) and two closed (50 × 10 × 40 cm) arms elevated 40 cm above the floor with average light levels 40–55 lux. After placement in the center of the apparatus facing the closed arms, behavior was recorded for 5 min (with the camera focused on the rear 3/4th of the rat's body) and analyzed by Any-maze software (Stoelting Co., Wood Dale, IL). The percentage of open arm entries (number of entries into the open arms divided by total number of entries in both arms), time spent in the open arms, total distance traveled and total time spent immobile were noted. The anxiety index was calculated as described (32), where total exploration on the maze represents the total number of arm entries: Anxiety Index = 1- [[(time spent in open arms/total time on the maze)+(number of entries into open arms/total number of arm entries)]/2], where total time on the maze was 300 sec. Each animal was tested once.

### Euthanasia and Sample Collection

Rats were euthanized with Beuthanasia (0.22 mL/kg i.p., Schering-Plough Animal Health, Union NJ). Blood was withdrawn from the heart with an 18-gauge needle (between 15:00 and 17:00 h), placed in Eppendorf tubes and maintained at room temperature for 30 min. Blood samples were then centrifuged at 5,000 × g at 4°C for 5 min, the serum was collected and stored at −80°C. CSF was withdrawn by inserting a 26-gauge needle into the cisterna magna and was immediately stored at −80°C. Brains and spinal cords were extracted and stored at −80°C. Brains were thawed on ice and sliced with a vibratome to dissect hippocampus, hypothalamus, amygdala, prefrontal cortex and periaqueductal gray (PAG) regions according to the Paxinos and Watson rat brain atlas (33) for N/OFQ and TNF-α mRNA and protein quantification.

### N/OFQ Quantification

N/OFQ content in sera, CSF and selected brain regions was determined by radioimmunoassay kit (Phoenix Pharmaceuticals, Belmont, CA) as previously described (10). Results are presented as N/OFQ; calculated and expressed in pg/mL for sera and CSF, and pg/mg for hypothalamus and PAG. All samples and standards were assayed in duplicate (50 μL). The sensitivity of the assay was 10 pg/mL and non-specific binding was 2.9%. There was no cross-reactivity with dynorphin A (1–17), enkephalin or β-endorphin.

### TNF-α

Blood samples were obtained by tail bleed (200–300 μL) under isoflurane anesthesia or from cardiac puncture. Serum and CSF was collected and prepared as described above. Levels of TNF-α in serum and CSF (50 μL) were quantified using the Rat TNF-α ELISA kit (KRC3011, Invitrogen) as directed by the manufacturer.

### Real-Time PCR

TRI reagent (Sigma-Aldrich, MO) was immediately added to dissected tissues for mRNA extraction. cDNA was synthesized using Super-Script III Reverse Transcriptase (Sigma-Aldrich, MO). Real-time PCR was performed using SYBR Green Master Mix (AnaSpec, Fremont, CA) and 125 nM forward and reverse primers (rat TNF-α FWD: 5′-ACCACGCTCTTCTGTCTACTG-3′, REV: 5′-CTTGGTGGTTTGCTACGAC-3′, rat 28S: FWD: 5′-GAAGGCAAGATGGGTCACCA-3′, REV: 5′-GAACTTCCGTGGGTGACTCC-3′, rat GAPDH Fwd: 5′-ACCCAGAAGACTGTGGATGG-3′, Rev: 5′-CAC ATT GGG GGT AGG AAC AC-3′, rat NOP Fwd: 5′-GTT CAA GGA CTG GGT GTT CAG CCA GGT AGT-3′, rat NOP Rev: 5′-TGC TGG CCG TGG TAC TGT CTC AGA ACT CTT-3′, rat preproN/OFQ Fwd: 5′-TGC ACC AGA ATG GTA ATG TG-3′, Rev: 5′-TAG CAA CAG GAT TGT GGT GA-3′, all from Sigma-Aldrich) in QuantStudio StepOne qPCR (Applied Biosystems). The average of GAPDH and 28S CT values served as an internal standard to which expression of other genes were normalized. Data were analyzed using the comparative Ct method as described (34).

### Blood Brain Barrier Permeability

Blood brain barrier permeability was determined by measuring the ratio of CSF albumin to serum albumin. Albumin levels in CSF (100 μL of a 1:500-fold diluted sample) and serum (100 μL of a 1:1,000,000-fold diluted sample) were collected from control or SPS-treated male rats euthanized at 1, 4, and 24 h (32 rats total) after being placed in isolation in their cages following recovery from ether anesthesia. Samples were analyzed by ELISA (GB0032, GenWay Biotech, Inc. San Diego, CA) based on manufacturer's instructions. The standard curve ranged from 0 to 200 ng/ml. Levels of CSF albumin in four rats (three control, one 4 h SPS) were outside the range of detection. With an insufficient volume of CSF remaining to re-assay, the albumin CSF:serum ratio for those rats could not be determined.

### Statistical Analysis

Results of D'Agostino & Pearson omnibus normality tests determined if subsequent data analysis should utilize parametric or non-parametric approaches. Outliers were identified by ROUT. Data were analyzed by one- or two-way ANOVA with Tukey's *post-hoc* analysis, as indicated. Non-parametric data were analyzed using the Kruskal-Wallis test. Results were considered significantly different if *p* < 0.05. Analysis was performed with Prism v. 9.2 for Windows (GraphPad Software, Inc.).

## Results

### THL Prevents SPS-Induced Increase in Serum TNF-α

**Figure 1:**
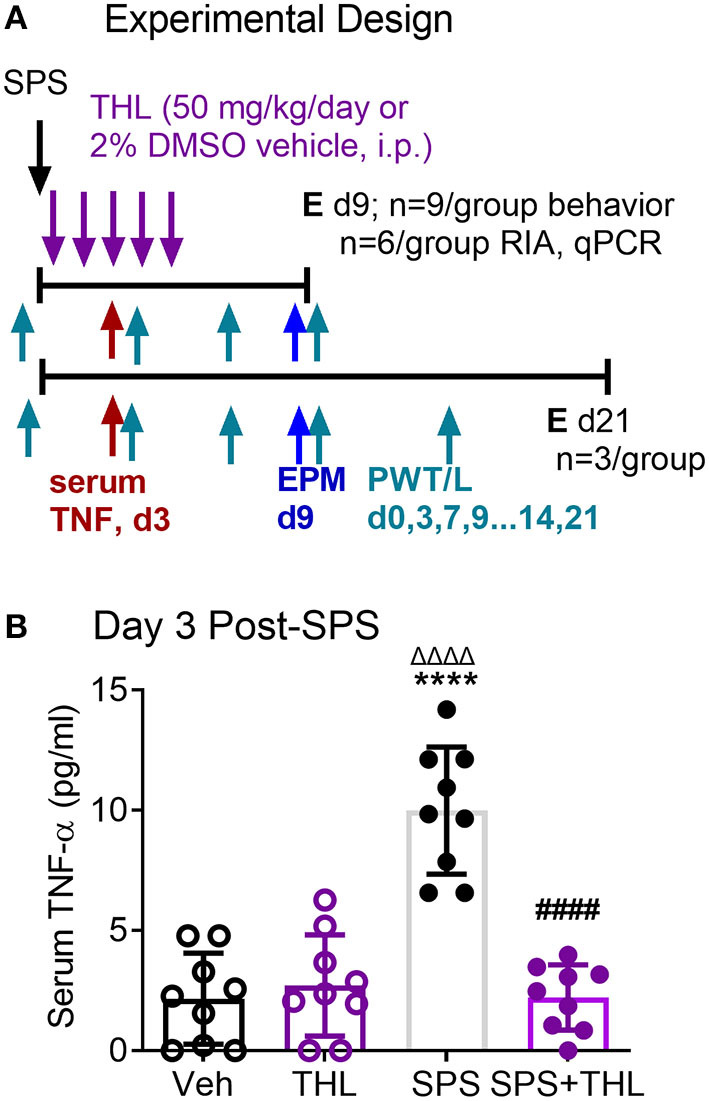
Thalidomide (THL) prevents TNF-α increase in serum of SPS rats. **(A)** Experimental design includes four groups [Vehicle control (Veh), thalidomide (THL), SPS+Veh (SPS) or SPS+THL], with 9 rats/group, 6 rats/group were euthanized (E) at day 9 and 3 in each group were euthanized at day 21. Nociceptive and anxiety-like behavior testing days are noted with arrows and the designations of PWT/L and EPM, respectively. **(B)** Blood was withdrawn from the tail vein on day 3 and serum THL levels were determined by ELISA. Data are presented as mean ± 95% confidence interval and were analyzed by 2-way ANOVA. Significant interaction between THL treatment and stress was noted [*F*_(1, 32)_ = 44.64, *P* < 0.0001], as well as significant effects of treatment and stress, *post-hoc* comparisons were made using Tukey's multiple comparison test (*p* < 0.0001 from Veh*****, THL^ΔΔΔΔ^, or SPS^####^).

We previously reported on preliminary studies that prior to appearance of anxiety-like behaviors at day 9, allodynia was evident as early as 3 days post-SPS, and was accompanied by elevated serum TNF-α levels in SPS-treated rats (35). Since that preliminary report, Sun et al. reported hyperalgesia within 1 day of SPS and increased TNF-α in hippocampus 1 day post-SPS (13, 14). As TNF-α can produce hyperalgesia, increase anxiety-like behaviors and increase N/OFQ expression, we hypothesized that blockade of the TNF-α surge would prevent or reduce the TNF-α increases and symptoms noted following exposure to SPS. Thalidomide (THL) blocks synthesis and release of TNF-α in the brain and periphery, so male rats subjected to SPS and their controls were treated with vehicle or an FDA-approved small molecule blood-brain barrier permeable TNF-α synthesis inhibitor (36). Vehicle control and SPS-treated rats received five single daily doses of THL or veh that were initiated immediately following SPS (designated day 1) and continued through day 5 (Figure 1A). To confirm the efficacy of THL treatment to inhibit TNF-α synthesis, blood samples were collected by tail bleed on day 3 of SPS. TNF-α levels in serum obtained from those samples was quantified by ELISA (Figure 1B) and analyzed by 2-Way ANOVA with Tukey's multiple comparisons test. Data analysis revealed a significant interaction between stress and THL treatment [*F*_(1, 32)_ = 44.64, *P* < 0.0001], as well as significant SPS [*F*_(1, 32)_ = 33.17, *P* < 0.0001] and treatment [*F*_(1, 32)_ = 35.75, *P* < 0.0001] effects. Treatment of SPS rats with THL prevented (#*p* < 0.0001) the TNF-α increase observed in SPS-treated rats (^*^p < 0.0001) 3 days post-SPS, but had no effect on TNF-α levels in in non-stressed rats (THL) compared to vehicle-treated controls (Veh), *N* = 9/group.

### THL Prevents Development of SPS-Induced Tactile Allodynia and Reduces Thermal Hyperalgesia

**Figure 2:**
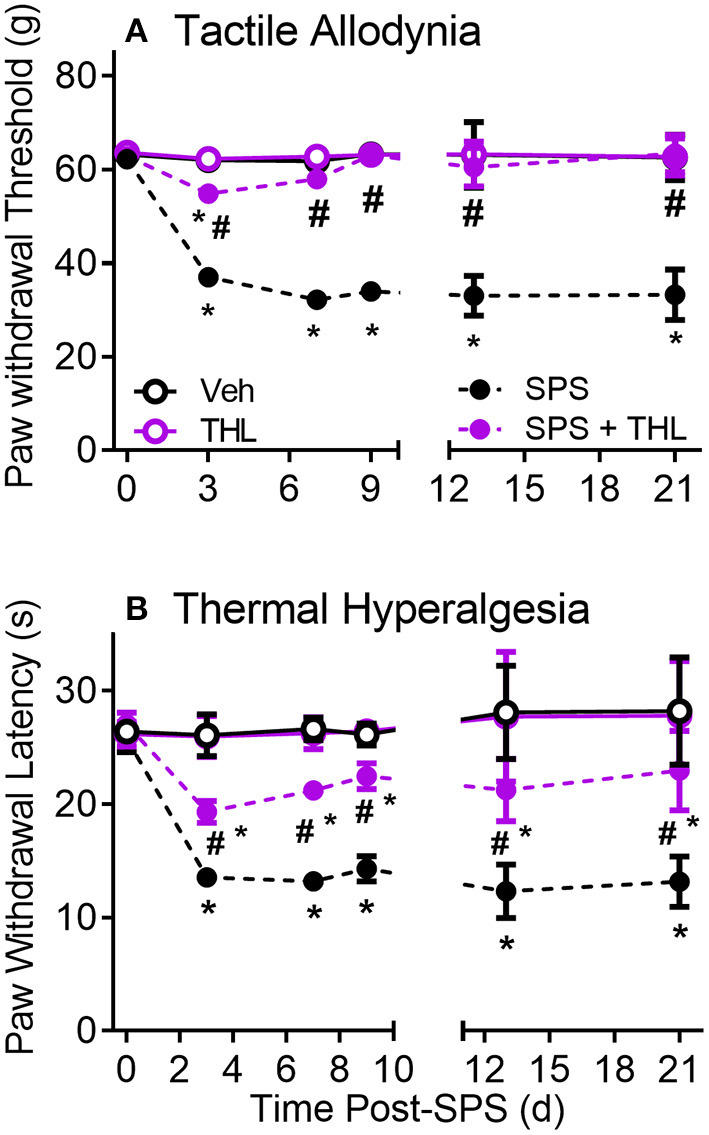
Thalidomide (THL) prevents development of SPS-induced tactile allodynia **(A)** and alleviates thermal hyperalgesia **(B)**. Single daily injections of thalidomide (50 mg/kg, i.p.) from days 1-5 of SPS prevents development of allodynia and reduces thermal hyperalgesia. Sensitivity of THL-treated rats did not differ from that of Veh-treated rats. Results reflect mean ± 95% CI of 9 rats/group through day 9 and 3 rats/group at days 13 and 21, group differs from Veh *p < 0.0001, differs from SPS: ^#^*p* < 0.001.

Nociceptive sensitivity to tactile (A) and thermal (B) stimuli was assessed prior to SPS (0) and on days 3, 7, and 9 (Figure 2, THL treatment = days 0–5). Data from day 0 to 9 were analyzed by a 2-Way ANOVA with repeated measures (*N* = 9/group) with Tukey's Multiple Comparison *post-hoc* test. PWT and PWL were significantly reduced in the SPS groups compared to Veh- and THL-treated groups (^*^p < 0.0001). Acute treatment of SPS with THL almost completely prevented the appearance of tactile allodynia (^#^*p* < 0.0001) compared to SPS on day 3, by day 7 no differences between SPS+THL, Veh- and THL-treated groups were noted (Figure 2A). THL treatment alone had no effect on baseline nociceptive sensitivity. THL also significantly alleviated thermal hyperalgesia in SPS rats (Figure 2B), however the effect of THL-treatment on thermal sensitivity was only partially effective as SPS+THL-treated rats continued to exhibit hyperalgesia (albeit less than SPS rats), throughout the 9 or 21 day periods (Figure 2B). Data analysis revealed a significant interaction between time and treatment for both tactile [*F*_(9, 96)_ = 306.4, *P* < 0.0001] and thermal [*F*_(9, 96)_ = 40.49, *P* < 0.0001] sensitivity. Significant effects of THL treatment on tactile [*F*_(3, 32)_ = 2912, *P* < 0.0001] and thermal sensitivity [*F*_(3, 32)_ = 250.5, *P* < 0.0001] and of time: tactile [*F*_(3, 96)_ = 494.2, *p* < 0.0001] and thermal sensitivity [*F*_(3, 96)_ = 99.98, *p* < 0.0001], were noted. To confirm that nociceptive sensitivity in THL-treated SPS rats did not gradually return to SPS levels over time, 3 rats per group also were assessed for nociceptive sensitivity on days 13 and 21 (Figure 2B). Sensitivity remained as it had been on day 9, with no changes over time. Two-way analysis of PWT and PWL for days 13 and 21 revealed a significant effect of treatment for tactile [*F*_(3, 8)_ = 274.9, *p* < 0.0001] and thermal [*F*_(3, 8)_ = 78.09, *p* < 0.0001] sensitivity.

### THL Prevents SPS-Induced N/OFQ Increases in Serum

**Figure 3:**
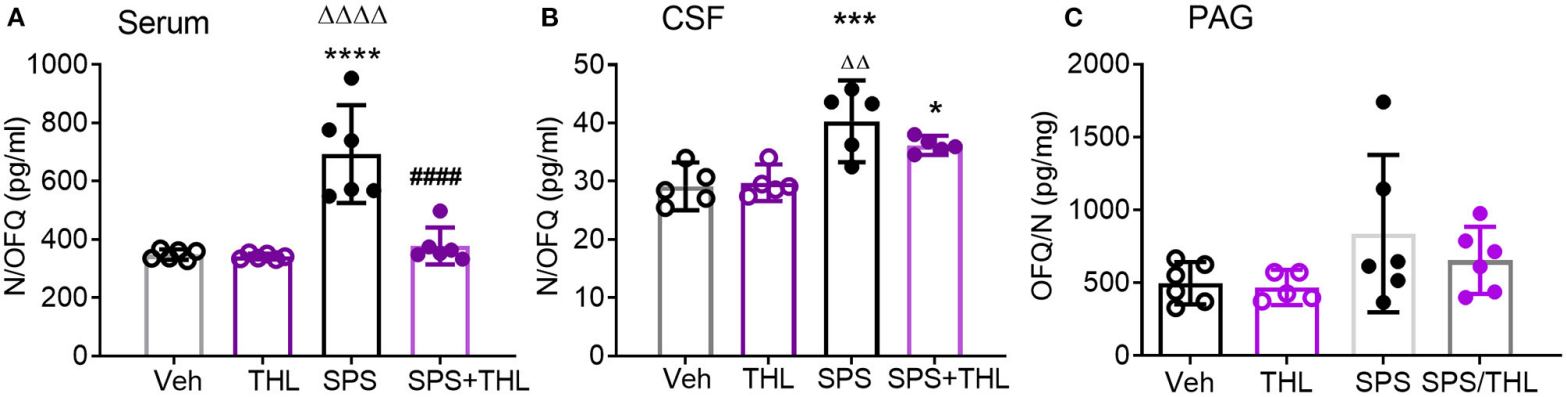
THL reduces SPS-induced increases in serum **(A)** and CSF N/OFQ **(B)**, but not PAG **(C)**. Data are presented as scatter plots with mean ± 95% CI. N/OFQ levels increased in serum (**A**, ****p < 0.0001) and CSF (**B**, ***p < 0.001) following SPS, THL treatment reversed N/OFQ increase in both serum and CSF (^####^*p* < 0.0001) as determined by two-way ANOVA with Tukey's multiple comparisons *post-hoc* tests. SPS also differed from THL (^ΔΔΔΔ^*p* < 0.0001, ^ΔΔ^*p* < 0.01). **(C)** A significant effect of SPS exposure on N/OFQ levels in PAG was observed [*F*_(1, 19)_ = 4.442, *p* = 0.04], but no *post-hoc* differences were found. Rout outlier test excluded one rat from THL **(C)**, *Q* = 1%.

The hypothesis that the early increase in serum TNF-α following SPS led to allodynia and hyperalgesia, as well as increases in central and circulating N/OFQ levels, was supported by a report that TNF-α increased N/OFQ expression (37). Therefore, the ability of acute THL treatment to prevent subsequent increases in N/OFQ post-SPS were determined in serum and CSF samples obtained from rats euthanized at day 9 and 21 post-SPS (Figure 3).

SPS increased serum N/OFQ levels compared to Veh- (^****^p < 0.0001) or THL treatment alone (^ΔΔΔΔ^*p* < 0.0001), and THL treatment blocked SPS-induced increases in serum N/OFQ (^####^*p* < 0.0001), as determined by two-way ANOVA (Figure 3A). There was a significant interaction effect between SPS and THL on serum N/OFQ levels [*F*_(1, 20)_ = 19.23, *P* = 0.0003]. Significant SPS [*F*_(1, 20)_ = 29.49, *P* < 0.0001] and treatment effects [*F*_(1, 20)_ = 20.92 *P* = 0.0002] also were observed.

Hemoglobin adulteration of CSF samples from 4 rats in the Veh-treated control group and 1 rat from each of the other 3 day 9 groups precluded assay. Since there were no differences between d9 and d21 vehicle-treated group CSF N/OFQ samples (as determined by unpaired student's *t*-test), they were grouped together to obtain sufficient sample size for analysis. A significant effect of stress was observed [Figure 3B, *F*_(1, 16)_ = 30.29, *p* = 0.0001]. SPS increased CSF N/OFQ levels compared to Veh- (^***^p < 0.001) and THL-treated groups (^ΔΔ^*p* < 0.01). Transient THL treatment lowered the SPS-induced increase in CSF N/OFQ levels, but SPS+THL still differed significantly from Veh (^*^p < 0.05). THL treatment did not alter baseline N/OFQ levels when compared to vehicle-treated controls from CSF or serum.

Since SPS also increased N/OFQ levels in the PAG at day 9-post SPS (35), N/OFQ-immunoreactivity was quantified in PAG from rats subjected to SPS in the presence and absence of Veh- or THL-treatments, and euthanized at day 9 (Figure 3C). There was a significant effect of SPS on PAG N/OFQ [*F*_(1, 19)_ = 4.442, *p* = 0.0486] as determined by two-way ANOVA (*N* = 5–6/group) (Figure 3C), but no *post-hoc* differences were found.

### SPS Reduced TNF-α mRNA in Hippocampus and Prefrontal Cortex

**Figure 4:**
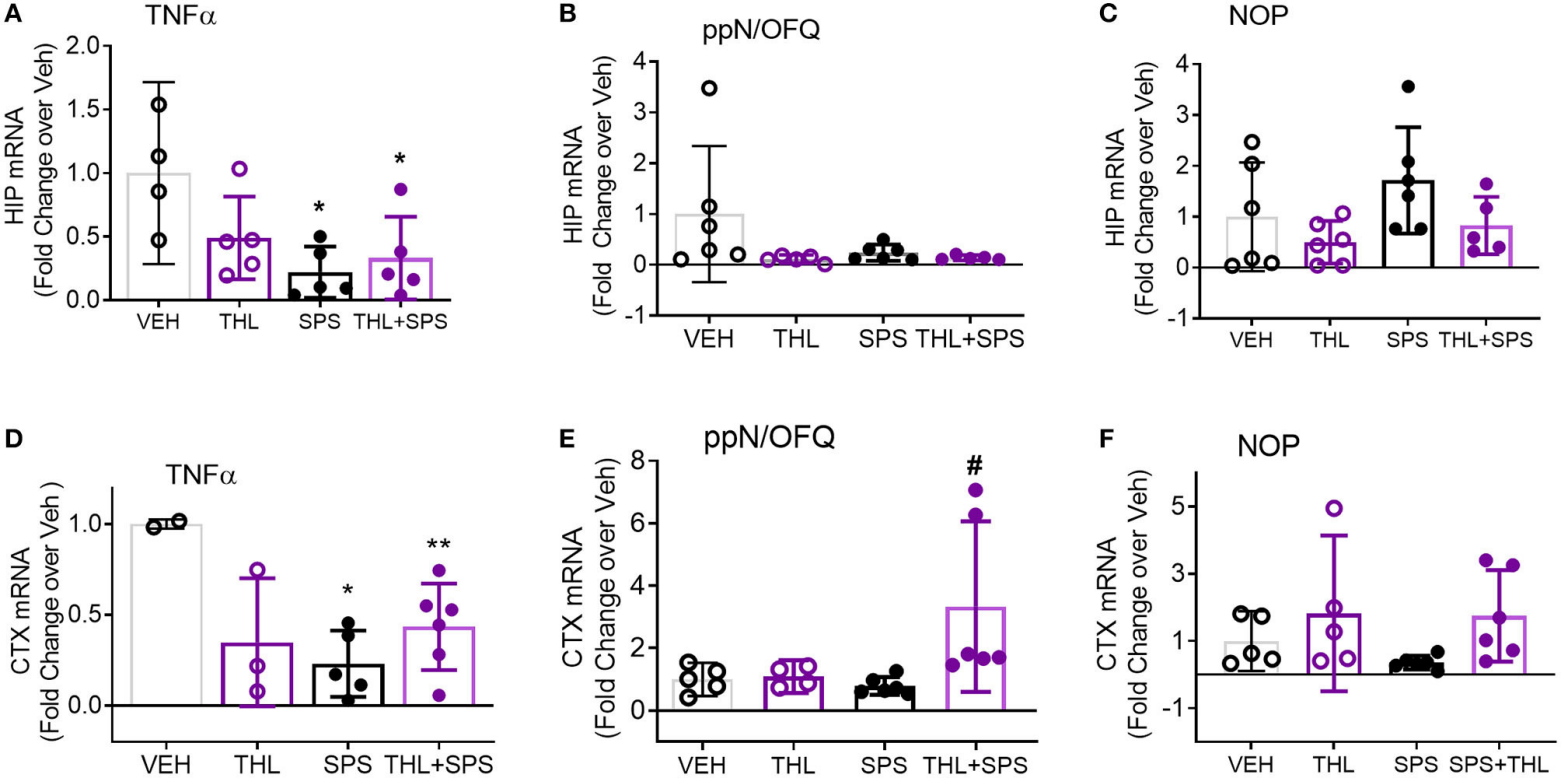
TNF-α mRNA expression declines in hippocampus (HIP) and prefrontal cortex (CTX) with SPS. Messenger RNA from HIP and CTX was isolated from tissues frozen immediately after extraction on day 9. Quantitative pcr (qPCR) was performed to determine changes in TNF-α, prepronociceptin (ppN/OFQ), and NOP receptor mRNA expression in all four groups. The ratio of each sample to GAPDH/28S was normalized to the mean of vehicle control to determine fold change in mRNA with treatment. Data were analyzed by one-way ANOVA with Tukey's multiple comparisons test **(A–C,E)** or Kruskal-Wallis **(B,F)**. Purity of mRNA from one Veh and one THL rat were below the threshold for use (0.8). Rout outlier test exclusions include 1 THL+SPS **(A)**, 1 SPS and 1 THL+SPS **(B)**, 1 THL+SPS **(C)**, and 1 THL **(E)**. TNF message was below the level of detection for 1 Veh **(A)**, and 3 Veh, 2 THL, and 1 SPS **(D)**.

The hippocampus (HC), amygdala (AMY), and prefrontal cortex (PFC) process responses to traumatic stress and pain, and express both the NOP receptor and N/OFQ. N/OFQ mRNA in PAG and HC, and NOP mRNA in AMY and PAG were elevated at day 21 post-SPS (17), but earlier time points have not been assayed. Messenger RNA from HC, AMY, and PFC was isolated from rats in Veh, THL, SPS, and SPS+THL groups euthanized at day 9 post-SPS. Changes in TNF-α, prepronociceptin and NOP receptor mRNA expression in those brain regions were determined using qPCR (Figures 4A–F). TNF-α mRNA levels were reduced in SPS and SPS+THL groups in HIP [Figure 4A: ^*^p < 0.05 by *F*_(3, 15)_ = 4.705, *p* = 0.0165] and prefrontal cortex [CTX, Figure 4D: ^*^p < 0.05, ^**^p < 0.01, *F*_(3, 12)_ = 5.206, *p* = 0.0156] compared to VEH. Decreased TNF-α mRNA is consistent with compensatory down-regulation following gene activation. Serum and CSF samples from day 9 rats also were assayed for TNF-α levels by ELISA, using a 2-way ANOVA. There was a significant difference between groups for CSF *F*_(1, 16)_ = 8.960, *p* = 0.0086, but no *post-hoc* differences were noted. No significant differences in TNF-α between treatment groups for serum were found (data not shown).

Though no differences were noted for N/OFQ in HIP (Figure 4B), a significant difference between groups was noted with N/OFQ mRNA for prefrontal cortex (Figure 4E), [*F*_(3, 17)_ = 3.969, *p* = 0.0259], with an increase in PNOC noted in the THL+SPS group compared to Veh alone (#*p* < 0.05).

No changes in NOP receptor mRNA were noted in HIP (Figure 4C). Kruskal-Wallis analysis found a significant difference in group means (Figure 4F, ^*^p = 0.0351), but no *post-hoc* differences were found. No changes in TNF-α, PNOC or NOP receptor mRNA were found in AMY (data not shown). These changes are consistent with differential regional modulation of NOP receptor peptide and receptor by TNF-α.

### THL Effects on Anxiety-Like Behaviors

**Figure 5:**
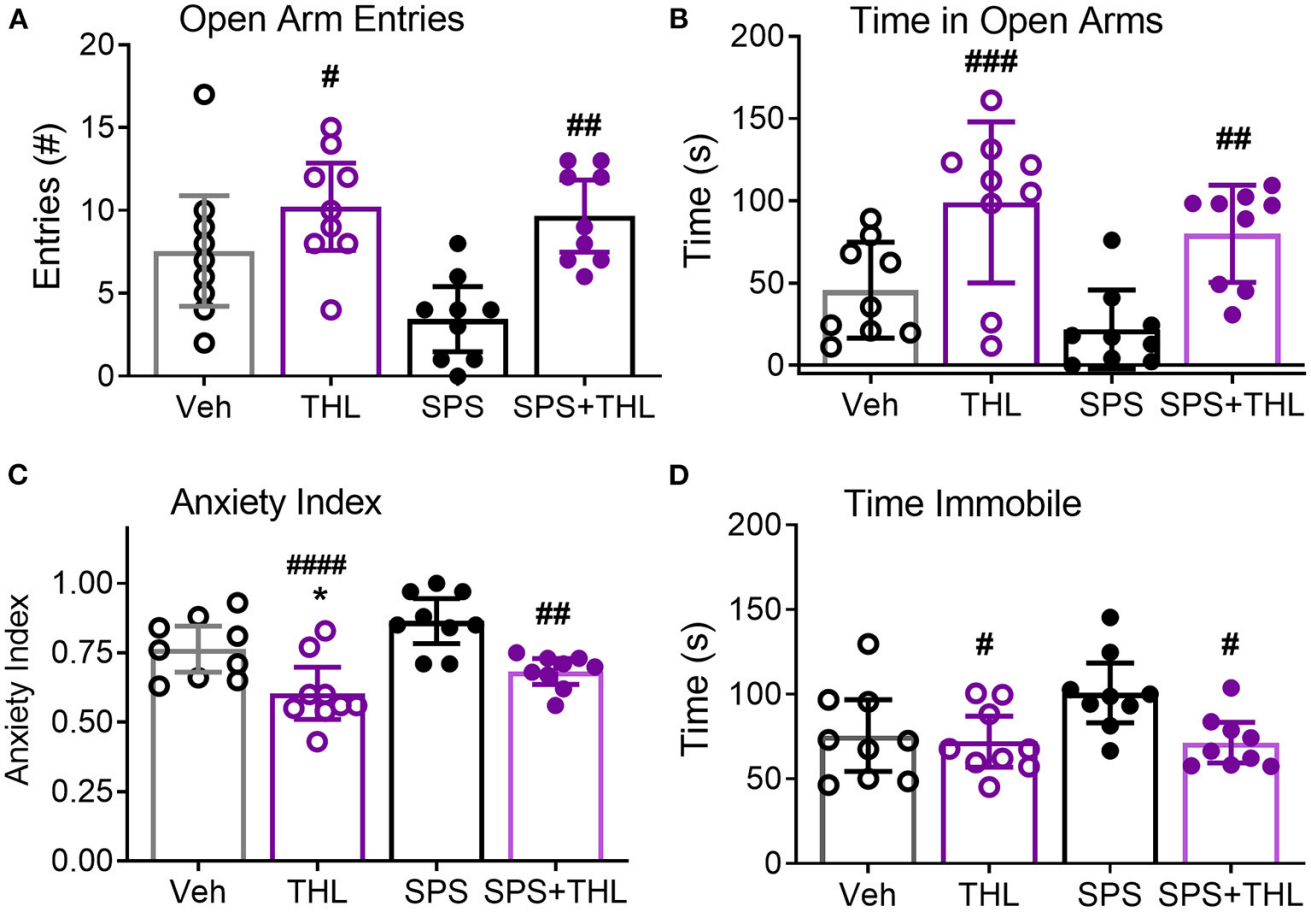
THL modulates anxiety-like behaviors in vehicle-treated control and SPS rats. Results of four different parameters are shown: Open arm entries **(A)**, Time in open arms **(B)**, Anxiety index **(C)**, and Time immobile **(D)**. Data are represented as scatter plots of *N* = 9/group, with 95% CI. *Post-hoc* analysis indicates significant effects of THL treatment alone: differs from Veh-treated (*p < 0.05) and SPS (^#^*p* < 0.05, ^##^*p* < 0.01, ^###^*p* < 0.001, and ^####^*p* < 0.0001.).

Since acute THL treatment prevented SPS-induced increases in serum TNF-α, nociceptive sensitivity and modulated NOP receptor and peptide mRNA, its effect on the development of anxiety-like behaviors was assessed in Veh-, THL-, SPS-, and SPS+THL-treated rats using the EPM test (Figure 5). SPS was a significant factor in decreased number of open arm entries and in the increased anxiety index (Table 1), consistent with previous studies (10, 17). However, *post-hoc* analyses did not confirm that SPS groups differed from Veh-treated controls in any parameter shown. THL treatment was a significant factor in *all* parameters assessed (Table 1) and *post-hoc* tests revealed that rats receiving THL-treatment alone differed from Vehicle- (^*^p < 0.05) or SPS-treated rats (#*p* < 0.05), making it difficult to interpret the impact of THL treatment on anxiety-like symptoms produced by SPS.

**Table 1:** ANOVA results of anxiety-like behaviors from THL and anti-TNF-α antibody treatment studies using EPM.

**Parameter**	**Factor**	***F*** **_(DFn, DFd)_**	***P*** **-value**
**Thalidomide-SPS EPM**			
Open arm entries	Interaction	*F*_(1, 32)_ = 2.528	0.1216
	SPS	*F*_(1, 32)_ = 4.356	**0.0449**
	THL	*F*_(1, 32)_ = 15.84	**0.0004**
Time in open arms	Interaction	*F*_(1, 32)_ = 0.04559	0.8323
	SPS	*F*_(1, 32)_ = 3.530	0.0694
	THL	*F*_(1, 32)_ = 23.83	**<0.0001**
Anxiety index	Interaction	*F*_(1, 32)_ = 0.1070	0.7457
	SPS	*F*_(1, 32)_ = 7.021	**0.0124**
	THL	*F*_(1, 32)_ = 25.05	**<0.0001**
Time immobile	Interaction	*F*_(1, 32)_ = 3.137	0.0860
	SPS	*F*_(1, 32)_ = 2.847	0.1012
	THL	*F*_(1, 32)_ = 5.103	**0.0308**
**α-TNFα Treatment—EPM**	**Sex/Panel**		
Open arm time	Males (A)	*F*_(2, 18)_ = 8.366	**0.0027**
	Females (D)	*F*_(2, 19)_ = 2.319	0.1255
Anxiety index	Males (B)	*F*_(2, 19)_ = 6.494	**0.0071**
	Females (E)	*F*_(2, 20)_ = 0.7449	0.4875
Time immobile	Males (C)	*F*_(2, 19)_ = 0.7839	0.4708
	Females (F)	*F*_(2, 20)_ = 0.5612	0.5793

*Bold font represents that the factor was significant in the thalidomide-SPS study (Figure 4), or that there was a significant difference between groups for each of the panels in the anti-TNF-alpha SPS study (Figure 11)*.

### BBB Permeability 1–24 h Post-SPS

**Figure 6:**
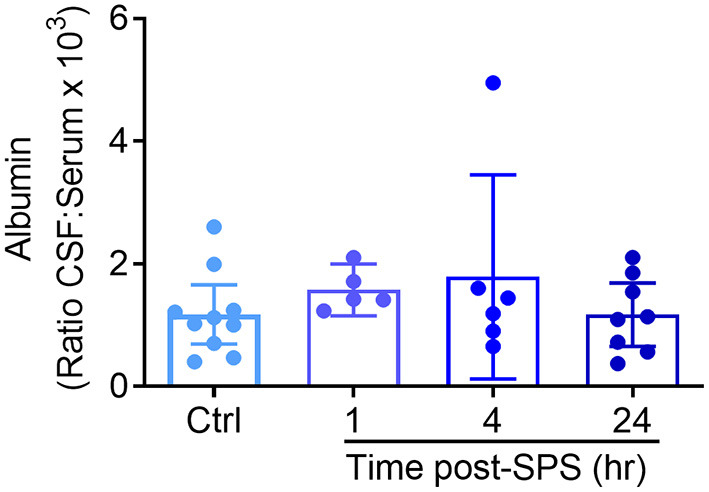
No evidence that SPS disrupts blood brain barrier permeability between 1 and 24 h post-SPS. The ratio of albumin in the CSF and serum from rats euthanized at each time point post-SPS was determined by ELISA and is presented as a scatter plot with mean ± 95% CI. No differences in the CSF to serum albumin ratio were found between groups (*p* = 0.3571) by Kruskal-Wallis test.

The blood-brain barrier (BBB) acts a selective physical barrier between the CNS and the periphery whereby it regulates the transport of molecules between both compartments (38). While albumin is present in high amounts in serum, it is normally excluded from CSF due to its high molecular weight. BBB disruption in humans is often measured by determining the ratio of albumin in a subject's CSF compared to levels in serum (Albumin_CSF:serum_ ratio) (39). Elevated ratios indicate BBB disruption (40–43). Traumatic stress was found to produce acute, transient increases in BBB permeability (44). Therefore, to determine if SPS alters BBB permeability, CSF and serum samples were recovered from SPS or control rats euthanized at 1, 4, and 24 h following recovery from ether anesthesia (Figure 6), and albumin levels determined by ELISA. Data from control rats from each time point comprised the control group. Levels of CSF albumin in three control and one 4-h SPS-treated rats were outside the level of detection, thus the CSF:serum ratio for those rats could not be determined. One rat from the 4 h group was determined to be an outlier by the ROUT (*Q* = 1%), and was excluded. Because three of the four groups failed the Shapiro & Wilk normality test, analysis was performed using the Kruskal-Wallis test. No significant differences between groups were found, consistent with an intact BBB following SPS.

### Changes in Circulating TNF-α and TNF-α mRNA 1–24 h Post-SPS

**Figure 7:**
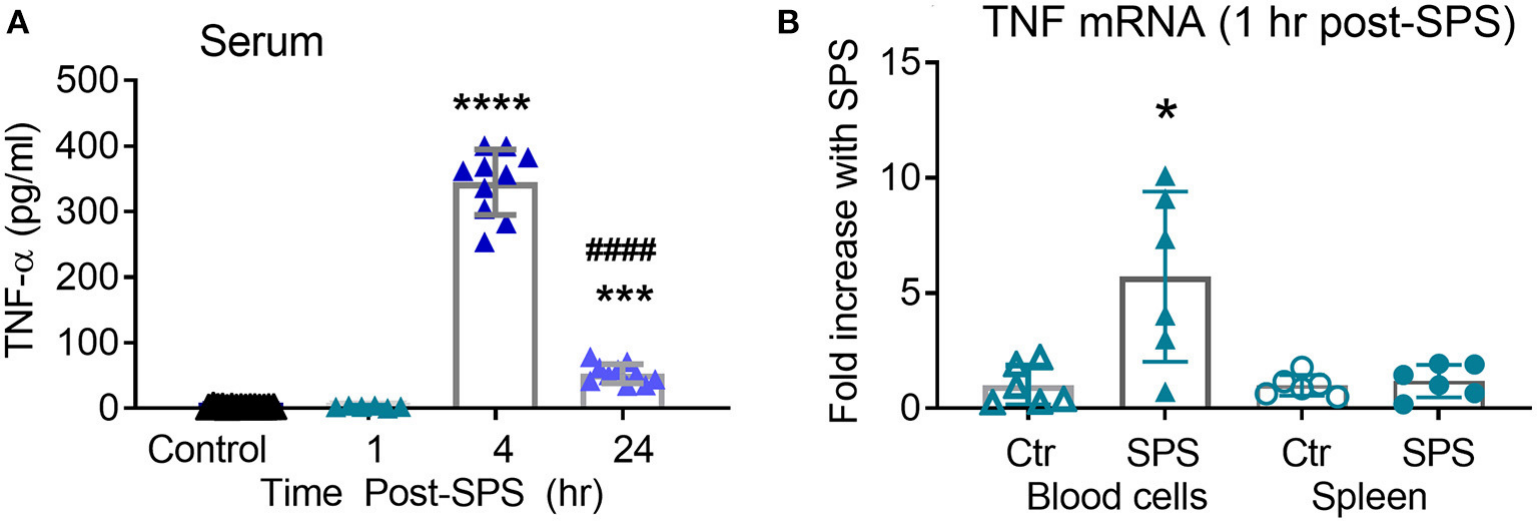
Serum TNF-α increases 4–24 h post-SPS. Serum samples collected for blood brain barrier permeability also were assayed for TNF-α levels by ELISA **(A)**, data presented as scatter plot with mean ± 95% CI (*N* = 6–16/group). TNF-α mRNA **(B)** increased in circulating blood cells (*p* < 0.05), but not spleen, 1 h post-SPS as determined by unpaired student's *t*-test. Real-time qPCR was performed as described above, normalized to mean 2^ΔCT^ of control rats to get fold change (*n* = 6 per group).

While levels of TNF-α in serum at 3 days post-SPS were higher than in untreated rats, they were still lower than would be expected immediately following exposure to a traumatic stress. Serum TNF-α often increases shortly after an injury or stressful event, and it was likely that the day 3 time point represented the trailing end of an earlier surge in TNF-α levels. Thus, serum TNF-α from rats in Ctrl, 1, 4, and 24 h post-SPS groups was determined by ELISA (Figure 7A). As anticipated, a significant difference between groups was noted [*F*_(2, 27)_ = 379.3, *P* < 0.0001] using one-way ANOVA with Tukey's multiple comparison test. Serum TNF-α levels increased 4 and 24 h after SPS compared to the control group (^***^p < 0.001, ^****^p < 0.0001). The 24 h post-SPS group also differed significantly from the 4 h post-SPS group (^####^*p* < 0.0001), confirming that levels began to decline after 4 hr. Both 4 and 24 h time points (Figure 7A) were higher than levels noted 3 days post-SPS (Figure 1B). TNF-α levels were not elevated in the serum at 1 h, therefore TNF-α mRNA levels were quantified from two potential tissue sources of circulating TNF-α (peripheral blood cells and spleen), 1 h post-SPS (Figure 7B). TNF-α mRNA levels did not differ in spleen between groups, but TNF-α mRNA increased 5-fold in circulating blood cells isolated from SPS rats (^*^p < 0.05) as determined by unpaired *t*-test.

### Effects of Anti-TNF-α Antibody Treatment on Development of Allodynia and Hyperalgesia in Male and Female Rats Following SPS

**Figure 8:**
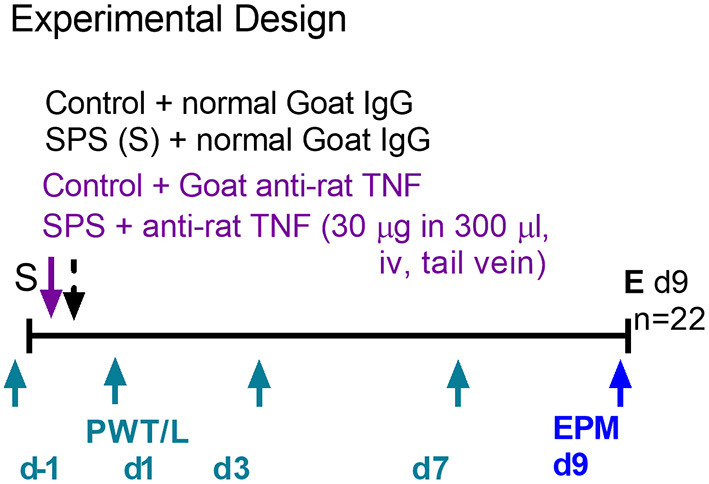
Experimental design for anti-TNF-α antibody treatment in males. Female design was the same except nociceptive sensitivity assessments also were made on d9 (*n* = 24 for female rats).

To support the hypothesis that circulating TNF-α released 1–4 h post-SPS resulted in SPS-induced allodynia, hyperalgesia and anxiety-like behaviors, male and female SD rats were divided into one of four groups: Sham Control + normal rat serum (Ctr/IgG), Sham Ctr + anti-TNF-α antibody (Ctr/α-TNF-α), SPS+IgG and SPS + α-TNF-α. Three hundred μl (30 μg) IgG or anti-TNF-α was injected into the tail vein (i.v.) during the last few min of the 2 h period of restraint in step one of the SPS protocol (Figure 8). Four hour post-SPS, rats were anesthetized with isoflurane and ~250 μl of blood volume was withdrawn from the tail vein to assay for serum TNF-α. Unfortunately, none of the assays (males or females) detected serum TNF-α (2, 4, or 24 h), perhaps because the IgG interfered with the assay.

**Figure 9:**
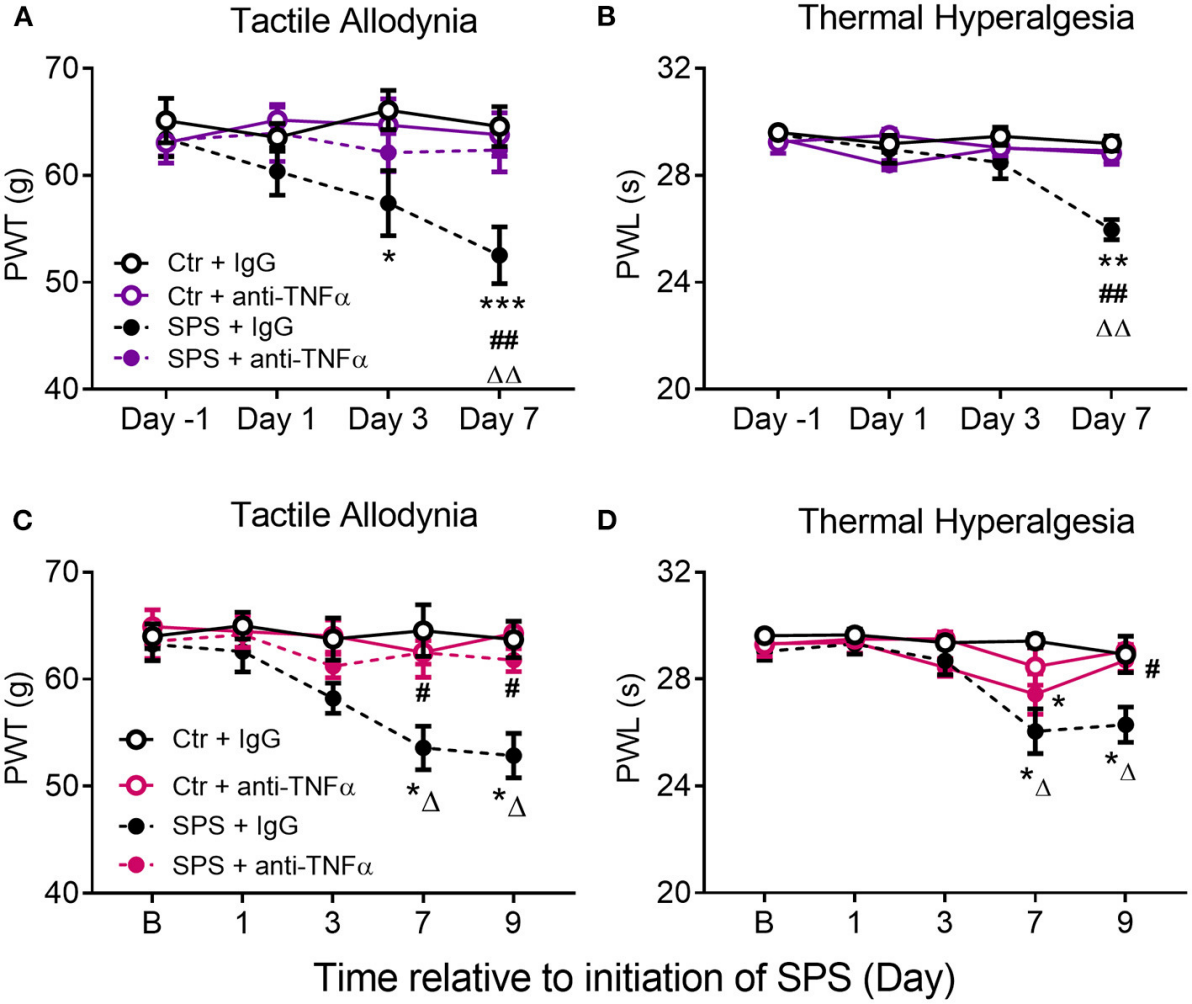
Anti-TNF-α antibody treatment prevents development of SPS-induced tactile allodynia **(A,C)** and thermal hyperalgesia **(B,D)** in male **(A,B)** and female **(C,D)** rats. Rats received a single injection of goat-anti rat TNF-α diluted in saline or the same volume of goat IgG during the SPS procedure. Data are presented as mean ± SEM of 5–6 rats per group. Data were analyzed by two-way ANOVA with Tukey's multiple comparison test. Significant differences were noted as follows: from control: ^*^p < 0.05, ^**^p < 0.01, ^***^p < 0.001; from Ctr+anti-TNFα: ^ΔΔ^*p* < 0.01, ^ΔΔΔ^*p* < 0.001; and from SPS: ^#^*p* < 0.05, ^##^*p* < 0.01.

Assessment of sensitivity to tactile and thermal stimuli occurred on days 1, 3, 7, and 9 (for females) post-SPS, and assessment of anxiety-like behaviors took place on the morning of day 9, rats were euthanized on day 9 following EPM assessment. As anticipated, SPS produced tactile allodynia and thermal hyperalgesia in males (Figures 9A,B, purple symbols) and females (Figures 9C,D, fuchsia symbols). Treatment of SPS rats with the anti-TNF-α antibody (SPS+α-TNF-α antibody) protected them almost completely from developing allodynia and hyperalgesia, similar to rats receiving THL. Data were analyzed by 2-way ANOVA with Tukey's multiple comparisons test, revealing a significant effect of anti-TNF antibody treatment on tactile allodynia [*F*_(3, 72)_ = 7.640, *p* = 0.0002] and a significant interaction between treatment and time for thermal hyperalgesia in males [*F*_(9, 72)_ = 4.753, *p* < 0.0001]. Significant effects of time and treatment also were noted for thermal hyperalgesia in males. For females, data analysis revealed significant interactions between anti-TNF-α antibody treatment and time for tactile allodynia [*F*_(12, 98)_ = 1.897, *p* = 0.0437] and thermal hyperalgesia [*F*_(12, 98)_ = 2.224, *p* = 0.0160]. Increased sensitivity in an SPS+anti-TNF-α group was noted only to thermal stimuli in female rats at day 7, but it was back to baseline by day 9 (Figure 9D). Therefore, the single injection of anti-TNF-α antibody shortly after traumatic stress prevented development of allodynia and hyperalgesia in male and female rats.

### Effects of Anti-TNF-α Antibody Treatment on N/OFQ Levels in Male and Female Rat Brain and Serum

**Figure 10:**
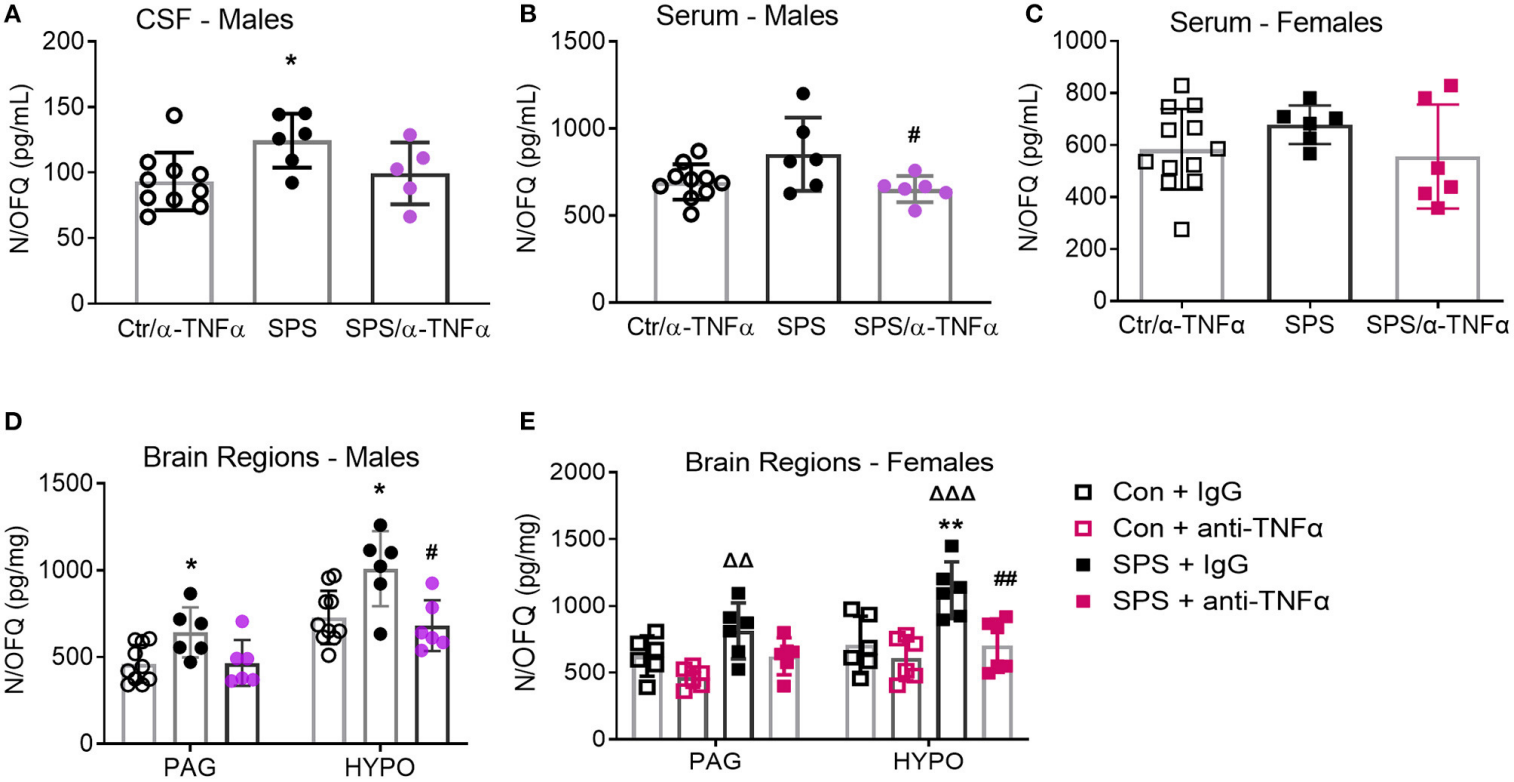
Anti-TNF-α antibody treatment prevents SPS-induced increases in N/OFQ levels in males and females. Data from sham controls receiving IgG and sham controls receiving anti-TNFα antibody injections were combined and compared to SPS+IgG and SPS+anti-TNF-α antibody groups collected on day 9 post-SPS as scatter plots showing mean ± 95% CI. One SPS+anti-TNF antibody rat sample was excluded by Rout **(A)**. SPS treatment increased N/OFQ levels in CSF **(A)**, periaqueductal gray (PAG: **D**) and hypothalamus (HYPO: **D**) in male rats, *p < 0.05 as determined by one-way ANOVA compared to sham-treated rats. Antibody treatment prevented N/OFQ increases in the HYPO (^#^*p* < 0.05). Though N/OFQ levels were not elevated by SPS in serum **(B)**, N/OFQ levels were significantly lower in serum from SPS + α-TNF-α antibody treated rats than from SPS rats (^#^*p* < 0.05). There were no significant differences of serum N/OFQ between groups in females **(C)**. However, SPS-induced increases in female PAG and HYP (^ΔΔ^*p* < 0.01 and ^ΔΔΔ^*p* < 0.001 from Con+anti-TNFα), and anti-TNFα antibody treatment reversal of increased N/OFQ in HYP (^##^*p* < 0.01 compared to SPS) **(E)** were the same as those in males **(D)**.

There were no differences in N/OFQ levels between Ctr+IgG and Sham+α-TNF-α, so data from those groups were pooled (designated as Ctr/α-TNF-α) for male and female serum (Figures 10A–D). SPS increased N/OFQ levels in male CSF [*F*_(2, 18)_ = 3.90, *p* = 0.0392], PAG [*F*_(2, 19)_ = 4.522, *p* = 0.0248] and hypothalamus [*F*_(2, 19)_ = 6.853, *p* = 0.0057] post-SPS as determined by one-way ANOVA (Figures 10A,C). Since TNF-α can increase N/OFQ mRNA and peptide, we posited that increased TNF-α with SPS leads to increased synthesis and release of N/OFQ that contributes to SPS-induced allodynia, hyperalgesia and anxiety-like behaviors. One-way ANOVA reveals that the anti-TNF-α antibody treatment prevented increased N/OFQ levels in serum (^#^*p* < 0.05), and HYPO in males (^#^*p* < 0.05) (Figures 10B,D). CSF samples are more difficult to obtain from female rats. Unfortunately, all CSF samples assayed from female rats were outside the range of the RIA kit except for SPS, and there was insufficient volume of CSF to repeat the assay. No differences between treatment groups were noted with serum from female rats collected at day 9 (Figure 10D, *p* = 0.2203).

However, PAG and HYP from female rats exhibited the same SPS- and α-TNFα treatment induced changes in N/OFQ as males (Figure 10E). Female PAG Ctr and α-TNFα alone groups did differ by *t*-test, so those groups were not pooled for either brain region in Figure 10E. For PAG, there was a significant effect of SPS [*F*_(1, 20)_ = 8.507, *p* = 0.0085] and of α-TNFα treatment [*F*_(1, 20)_ = 8.706, *p* = 0.0079]. N/OFQ levels in PAG from SPS rats differed significantly from Ctr + α-TNFα (^ΔΔ^*p* = 0.01). For female hypothalamic N/OFQ, there was a significant effect of SPS [*F*_(1, 20)_ = 10.45, *p* = 0.0042] and of α-TNFα [*F*_(1, 20)_ = 10.92, *p* = 0.0035]. SPS differed from control (^**^p < 0.01), Ctr + α-TNFα (^ΔΔΔ^*p* < 0.001), and from SPS + α-TNFα (^##^*p* < 0.01).

Comparisons between male and female N/OFQ for serum, PAG and HYP were performed by 2 way ANOVA. For serum N/OFQ, no interaction between sex and treatment group was found, but there were significant effects of sex [*F*_(1, 40)_ = 8.139, *p* = 0.0068] and of group [*F*_(2, 40)_ = 4.325, *p* = 0.0199]. No *post-hoc* differences between males and females were noted within any treatment group. Similar results were obtained when analyzing N/OFQ levels in the PAG, with effects of sex [*F*_(3, 38)_ = 9.539, *p* = 0.0037] and group [*F*_(3, 38)_ = 7.466, *p* = 0.0005] but no differences between males and females within each group. In the hypothalamus, no differences were noted with sex as a variable, but there was a significant group effect [*F*_(3, 38)_ = 12.14, *p* < 0.0001].

### Effects of Anti-TNF-α Antibody Treatment on N/OFQ and NOP Receptor mRNA in Male and Female Rat Brain Regions

Levels of ppN/OFQ and NOP receptor mRNA from HYP, PAG, prefrontal cortex (CTX) and amygdala in male and female rats treated with or without anti-TNF-α antibody and SPS were quantified by qPCR to determine if circulating TNF-α contributes to changes noted with SPS. SPS-induced increases in ppN/OFQ mRNA in the female PAG (Table 2) that paralleled increased N/OFQ peptide in that brain region (Figure 10E), but unlike the peptide, α-TNFα treatment reversed SPS-induced 2.4-fold increase in ppN/OFQ mRNA levels (^##^*p* < 0.01). Similar effects were found with ppN/OFQ mRNA in female CTX (reversal of 1.8-fold mRNA increase). In the male HYP, SPS + α-TNFα treatment significantly increased ppN/OFQ mRNA compared to SPS (^#^*p* < 0.05). Despite increased N/OFQ peptide in male PAG with SPS (Figure 10D), ppN/OFQ mRNA levels did not differ from controls in that region (Table 2). No other changes in N/OFQ mRNA were noted.

**Table 2:** Effects of α-TNF antibody treatment on SPS-induced changes in ppN/OFQ and NOP mRNA from Day 9 post-SPS and control rats.

	**ppN/OFQ mRNA (fold Change over Ctr)**			
**Group**	**Ctr**	**SPS/IgG**	**SPS/α-TNFα**	**ANOVA**
**Females**				
**Brain region**				
PAG	1.0 ± 0.5	**2.4** ± 1.2**	**1.1 ± 0.5^##^**	***P*** **= 0.0019**
HYP	1.0 ± 0.5	1.1 ± 0.6	0.8 ± 0.4	*P* = 0.6596
AMY	1.0 ± 0.7	0.5 ± 0.9	0.9 ± 0.5	*P* = 0.3986
CTX	1.0 ± 0.4	1.8 ± 1.0*	**1.0 ± 0.3^#^**	***P*** **= 0.0186**
**Males**				
**Brain region**				
PAG	1.0 ± 0.4	1.0 ± 0.6	1.0 ± 1.5	*P* = 0.9807
HYP	1.0 ± 0.8	0.1 ± 0.6	**1.8 ± 1.6^#^**	***P*** **= 0.0218**
AMY	1.0 ± 0.5	1.1 ± 0.3	1.2 ± 0.2	*P* = 0.7088
CTX	1.0 ± 1.2	1.4 ± 0.8	0.8 ± 0.3	*P* = 0.4961
	**NOP mRNA (fold change over Ctr)**			
**Group**	**Ctr**	**SPS/IgG**	**SPS/α-TNFα**	**ANOVA**
**Females**				
**Brain region**				
PAG	1.0 ± 0.4	1.9 ± 1.1*	1.2 ± 0.3	***P*** **= 0.0326**
HYP	1.0 ± 0.6	0.9 ± 0.4	1.0 ± 0.5	*P* = 0.9623
AMY	1.0 ± 0.6	0.9 ± 0.4	1.4 ± 0.6	*P* = 0.2156
CTX	1.0 ± 0.4	1.8 ± 1.3	1.5 ± 0.7	*P* = 0.1129
**Males**				
**Brain region**				
PAG	1.0 ± 0.3	3.3 ± 2.9	4.7 ± 6.0	*P* = 0.2475
HYP	1.0 ± 0.5	2.6 ± 0.9*	2.1 ± 1.5	***P*** **= 0.0167**
AMY	1.0 ± 1.2	0.6 ± 1.0	1.0 ± 1.0	*P* = 0.7886
CTX	1.0 ± 0.9	1.4 ± 1.0	0.7 ± 0.6	*P* = 0.4001

Individual values from SPS/IgG and SPS/α-TNFα groups (n = 6 each) were normalized to the mean value of combined Ctr/IgG and Ctr α-TNFα group (n = 12) to determine fold change in mRNA. Differences between groups were determined using one-way ANOVA with Tukey's multiple comparisons post-hoc test. Data are presented as mean ± SD, (*indicates difference from Ctr, # indicates difference from SPS). Bold font highlights significantly different groups.

SPS increased NOP receptor mRNA in PAG of female rats (^*^p < 0.05) and in the HYP of male rats (^*^p < 0.05), but this increase was not blocked by anti-TNF antibody treatment in either case (Table 2). No other changes in NOP mRNA were found in the other regions tested in males or females.

### Effects of Anti-TNF-α Antibody Treatment on SPS-Induced Anxiety-Like Behaviors in Male and Female Rats

**Figure 11:**
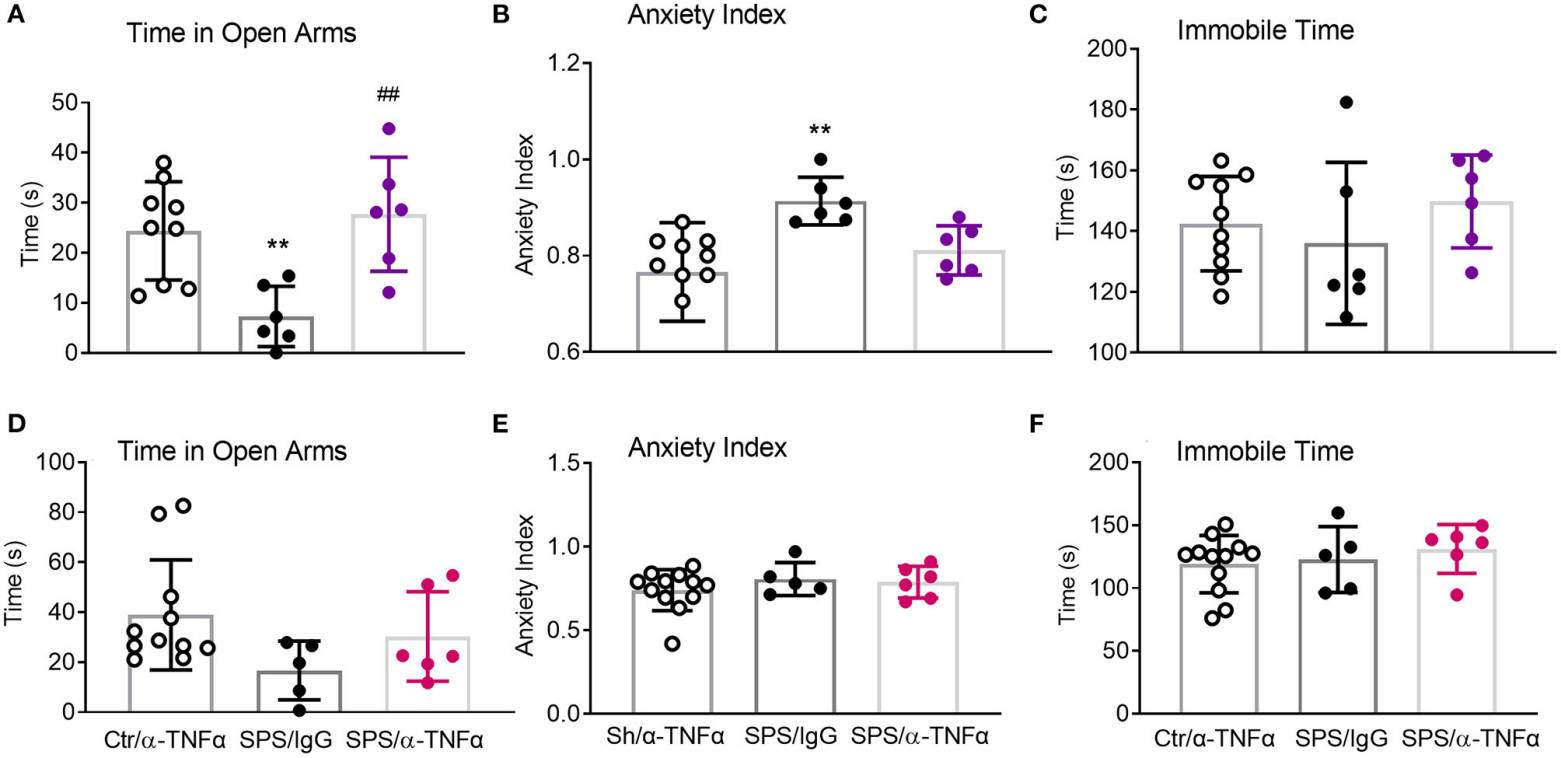
Anti-TNF-α antibody treatment had mixed effects on EPM parameters in males. Time in Open Arms **(A)**, Anxiety Index **(B)**, and Immobile time **(C)** were assessed in males **(A–C)** and females **(D–F)**, respectively. Data from sham controls receiving IgG and sham controls receiving anti-TNFα antibody injections were combined, and compared to SPS+IgG and SPS+α-TNF-α. SPS produced anxiety-like behaviors in male rats **(A,B)**, but not in female rats **(D,E)**. Anti-TNF-α antibody treatment reversed decreased time in open arms (**A**, ^##^p<0.01), but not the increased anxiety index **(B)**. Data are presented as scatter plots, showing mean ± SD.

Though not chosen as a primary endpoint, the robust nature of the traumatic stress on anxiety-like behaviors produced very interesting results (Figures 11A–F). As previously reported by others and us, SPS rats exhibited a number of anxiety-like behaviors 9 days post-SPS. Time in open arms was significantly reduced in SPS/IgG male rats (^**^p < 0.01, Figure 11A) and anxiety index increased (^**^p < 0.01; Figure 11B), consistent with elevated anxiety-like behaviors. Anti-TNF-α antibody treatment prevented the SPS-induced decreased time in open arms (##*p* < 0.01, Figure 11A), but did not quite reverse the elevated anxiety index, *p* = 0.09 (Figure 11B). No significant differences between groups were noted for any parameter for female rats (Figures 11D–F); no differences in immobile time were noted for males or females (Figures 11C,F).

## Discussion

This paper demonstrates, for the first time, the time course of circulating TNF-α following trauma in a preclinical model of PTSD (SPS). It also shows that that blockade of TNF-α synthesis or action prevented the development of SPS-induced allodynia and alleviated the development of hyperalgesia in male and female rats, as well as prevented upregulation of N/OFQ in serum of male rats, and in the HYP of male and female rats.

The pathophysiology of co-morbid PTSD and chronic pain is unclear. Patients with PTSD often exhibit excessive inflammatory activities of the immune system including increased circulating pro-inflammatory cytokines, however few studies have tested the relationship between inflammatory cytokines, PTSD and pain experimentally in preclinical models. Previous work in our lab and others has shown that the SPS preclinical model for PTSD induces the development of persistent allodynia, thermal hyperalgesia, visceral sensitivity and increased anxiety-like behaviors (10–14, 17, 18). Accompanying these exaggerated pain responses are increased N/OFQ levels in both serum and CSF, and an increase in circulating pro-inflammatory cytokine TNF-α 3 days after SPS. Elevated TNF-α is in concordance with human data whereby PTSD patients including those with non-combat related trauma were found to have elevated serum TNF-α compared to non-PTSD controls (21, 22). The frequency and severity of PTSD symptoms correlated with several different cytokines including TNF-α (21, 22). To ascertain whether the increase in serum TNF-α levels that occurs acutely post-SPS play a role in the development of SPS-induced nociceptive sensitivity and elevated N/OFQ, a TNF-α synthesis inhibitor, thalidomide, and an antibody targeting rat TNF-α were employed.

TNF-α may arise from peripheral and central sources, producing pro- and anti-inflammatory effects, including transcriptional activation. In the periphery, TNF-α may arise from several sources following severe stress such as spleen, lymph nodes, macrophages, NK cells, CD4+ lymphocytes, neutrophils, mast cells, and eosinophils (25, 45–52). We identified ~ 5-fold increase in TNF-α mRNA in circulating blood cells 1 h post-SPS (Figure 7), consistent with a peripheral TNF-α source acutely.

Besides neurons (19, 53), N/OFQ is expressed in glial cells from rat forebrain and SC, dorsal root ganglia (DRG) (54) and immune cells. Neutrophils secrete N/OFQ concurrent with degranulation, and N/OFQ induces leukocyte chemotaxis [for review see (55)]. The ability of N/OFQ to activate NFκB (56) provides a means for it to regulate cytokine and chemokine production as well.

Inflammatory mediators released by glial cells (TNF-α, CNTF) induce profound up-regulation of N/OFQ mRNA and peptide (19, 37, 53, 57). In the brain, N/OFQ acts as an “anti-opioid”, increasing hyperalgesia (58). Nanomole doses of N/OFQ produces analgesia when administered intrathecally (59), however femto-picomole levels of i.t. N/OFQ produce hyperalgesia and touch-evoked allodynia (60). Peripheral N/OFQ also contributes to hyperalgesia, distinct from the secondary hyperalgesic responses it may produce (61, 62). Up-regulation of N/OFQ mRNA in dorsal root ganglion and spinal cord following induction of experimental neuropathic pain was blocked with the microglial/peripheral immune cell inhibitor, minocycline (63). Minocycline also blocked PTSD fear and anxiety-like behaviors shortly after systemic (64) and intra-hippocampal administration (13), suggesting that the inflammatory mediator(s) mediating those actions, such as TNF-α, were produced in the hippocampus as well as peripherally. Spinal cord microglia and astrocytes are key players in pain modulation. They contribute to the initiation and maintenance of persistent pain states as well as provide structural and trophic support for neurons. Activation of microglia and astrocytes in the spinal cord contribute to central sensitization, characterized by the development of allodynia and hyperalgesia to tactile and thermal stimuli after nerve injury or peripheral inflammation (64–66).

Neuroinflammation in the CNS refers to the microglial response, and to a lesser extent that of astrocytes and oligodendrocytes (67). Although astrocytes and neurons are able to produce TNF-α, microglia are the primary source of this cytokine during neuroinflammation (67). During the initial response to acute stress, TNF-α activates corticotrophin releasing factor (CRF)-containing neurons in the hypothalamic paraventricular nucleus (68) resulting in CRF-mediated cortisol release, in order to induce negative feedback to prevent an overshoot of inflammatory processes (55, 69–72). PTSD chronically activates and dysregulates the HPA axis wherein negative feedback is enhanced (73).

The ratio of albumin in CSF compared to serum serves as an index of the integrity of the blood-CSF (BBB) barrier. Increases in this ratio denote increased CSF permeability and thus, decreased BBB integrity. No changes in BBB permeability were detected over the 24 h period following SPS. This is consistent with a role of peripheral TNF-α in the initiation of allodynia and hyperalgesia. Though TNF-α may be transported across the BBB (74), increased BBB permeability would facilitate cytokine infiltration to the CNS. The presence of increased inflammatory mediators, such as TNF-α, leads to activation of microglia and recruitment of peripheral blood monocyte entry into the brain parenchyma that is associated with pain, depressive and anxiety disorders (75, 76).

Thalidomide crosses the BBB (77) to produce several centrally mediated effects (36, 78). Though we did not measure thalidomide levels, the significant effect of THL alone on anxiety-like behaviors, in the absence of traumatic stress, confirms that it penetrates the BBB. THL prevented the development of allodynia and reduced thermal sensitivity by 50%, even for at least 2 weeks after THL administration was discontinued (Figure 2). THL is not selective for TNF-α, but inhibits the synthesis of several inflammatory cytokines.

The efficacy of a single administration of a circulating antibody against TNF-α to prevent initiation of allodynia and almost completely alleviate hyperalgesia following SPS in the presence of an intact BBB suggests that the initial surge in serum TNF-α is an integral factor in initiating the pathophysiological cascade of events of SPS. This is in concordance with other experimental models implicating TNF-α with the emergence of pain symptoms and in particular the neuroinflammatory and nociceptive properties that play a role in central sensitization (79, 80). Serum TNF-α levels at 4 and 24 h post-SPS were significantly larger than day 3 post-SPS levels and were comparable to levels found in inflammatory disorders with depressive mood symptoms (81).

Antibodies in general cross the BBB very poorly unless the barrier is leaky to very large proteins, such as with chronic inflammation or brain tumor, or the antibody can target a receptor-mediated transport protein to do so (82). Our data indicates that the blood-brain barrier remained intact following SPS for at least 24 h following SPS (Figure 6), consistent with a previous study that examined a variety of stressful stimuli on blood-brain barrier integrity (83). If there was localized disruption following SPS, it was masked by the overall ability of the barrier to prevent albumin from entering the CNS. It is unlikely that significant amounts of the anti-TNF-α antibody crossed the BBB since the barrier appeared intact. Indeed previous use of an anti-TNF-α antibody (i.v.) to treat dental pain in rats, found that its ability to block pain was gone by the third day after i.v. administration (84). This limited time of action would suggest that allodynia and hyperalgesia in the SPS model would NOT have been alleviated on days 5–9 (as it was), if the peripheral source of the pain stimulation (TNF-α) had not been removed. Differences between reversal of N/OFQ levels in PAG and HYP by TNF antibody treatment also may reflect the relatively close proximity of the HYP to the BBB compared to the PAG. Further studies of changes in TNF and N/OFQ mRNA and peptide in those regions at earlier time points may provide clarification.

One limitation of the CSF:Serum albumin ratio method is that it does not directly measure the location and physical extent of BBB disruption, and small disruptions of the barrier may be masked. Additional methods may be useful to fully ascertain the sites and impact of stress/trauma on BBB integrity in this model in the future (85–88).

Of particular interest to this study is (1) previous evidence that TNF-α increased prepronociceptin mRNA and N/OFQ peptide (19), and that (2) N/OFQ activates NFκB through the NOP receptor (56). N/OFQ also appears to play a role in increased N/OFQ since blockade or loss of NOP prevents N/OFQ up-regulation at later time points as well (17, 18). Upregulated signaling by the neuropeptide Nociception/Orphanin FQ (N/OFQ)- NOP receptor complex downstream of TNF-α appears to sustain chronic pain and PTSD symptoms. N/OFQ levels are elevated in serum and cerebrospinal fluid (CSF) of patients with other forms of chronic pain, which is not surprising since supraspinal N/OFQ may increase pain sensitivity by inhibiting the descending analgesic pathway.

We previously reported that SPS increased levels of N/OFQ in the CSF and brain on days 9–28, and blockade or loss of NOP receptor prevented this increase in male rats. In the current study, serum N/OFQ levels also significantly increased by day 9 of SPS, but only in male rats. THL blocked increased N/OFQ in serum and CSF, correlating with alleviation of TNF-α-induced allodynia and hyperalgesia. Though the results with anti-TNF-α antibody treatment appear more nuanced, they reflect differences between males and females with SPS. We previously reported that female SPS rats did not show increased levels of N/OFQ in serum and CSF as the males did, and their anxiety-like symptoms were not evident at day 9 post-SPS (18), though allodynia and hyperalgesia were similar. However, SPS increased N/OFQ peptide in PAG and HYP of males and females at day 9, and only the N/OFQ increase in HYP was blocked by TNF-α antibody treatment. The 2-fold increase in ppN/OFQ mRNA in the PAG and prefrontal cortex in female rats after SPS is consistent with hyperalgesia and allodynia, even in the absence of increased N/OFQ in the serum. It was interesting that N/OFQ peptide levels in males increased in the HYP with SPS while NOP receptor mRNA also increased (Table 2). N/OFQ regulates the HPA axis primarily through its actions on the hypothalamus, so up-regulated disruption of peptide signaling would certainly be consistent with dysregulation of the N/OFQ-NOP system. Reversal of N/OFQ up-regulation in the hypothalamus by anti-TNF-α antibody treatment in males is consistent with alleviation of allodynia, hyperalgesia, and anxiety-like symptoms. Unlike what was reported for neuropathic pain, blockade of TNF-α action is therapeutic for female rats with traumatic stress-induced allodynia (89). However, in that study initiation of anti-TNF treatment was not begun until neuropathic pain was clearly established (1 wk after injury). Our study focused only on blocking TNF actions immediately after the trauma, before detection of allodynia. Pooley et al. (90) compared males and females in two different preclinical models of PTSD, including SPS. They found that in contrast to males, traumatic stress did not enhance negative feedback of the HPA axis in female rats. Clearly, much more work remains to understand the factors initiating traumatic stress and comorbid pain and anxiety-like behaviors, and the differences between males and females at both initiation and maintenance phases of pain and anxiety-like behaviors.

In summary, this study suggests that TNF-α is integral in the pathophysiology of PTSD leading to the development of SPS-induced allodynia and hyperalgesia, and modulates changes in N/OFQ peptide and transcript. Acute treatment with a short-acting, small molecule TNF-α blocking drug biological may offer a novel therapeutic method to prevent or reduce symptoms of PTSD and co-morbid pain.

## Data Availability Statement

The original contributions presented in the study are included in the article/supplementary material, further inquiries can be directed to the corresponding author/s. Some of the data was previously presented in poster form.

## Ethics Statement

The animal study was reviewed and approved by Institutional Animal Care and Use Committee of the University of Oklahoma Health Sciences Center and the US Army Medical Research and Materiel Command Animal Care and Use Review Office.

## Author Contributions

PD and YZ performed animal experiments and biochemical and molecular studies. PD, YZ, and KS analyzed the data. KS, PD, RG, and MI designed the experiments. PD and KS wrote the manuscript. All authors discussed and commented on the manuscript.

## Conflict of Interest

The authors declare that the research was conducted in the absence of any commercial or financial relationships that could be construed as a potential conflict of interest.

## Publisher's Note

All claims expressed in this article are solely those of the authors and do not necessarily represent those of their affiliated organizations, or those of the publisher, the editors and the reviewers. Any product that may be evaluated in this article, or claim that may be made by its manufacturer, is not guaranteed or endorsed by the publisher.
